# Tongue Image Database Construction Based on the Expert Opinions: Assessment for Individual Agreement and Methods for Expert Selection

**DOI:** 10.1155/2018/8491057

**Published:** 2018-10-02

**Authors:** Zhen Qi, Li-ping Tu, Zhi-yu Luo, Xiao-juan Hu, Ling-zhi Zeng, Wen Jiao, Xu-xiang Ma, Cong-cong Jing, Wei-jian Wang, Zhi-feng Zhang, Jia-tuo Xu

**Affiliations:** ^1^Basic Medical College, Shanghai University of Traditional Chinese Medicine, 1200 Cailun Road, Shanghai 201203, China; ^2^Shanghai Collaborative Innovation Center of Health Service in Traditional Chinese Medicine, Shanghai University of Traditional Chinese Medicine, 1200 Cailun Road, Shanghai 201203, China; ^3^Physical Examination Center, Shuguang Hospital Affiliated to Shanghai University of Traditional Chinese Medicine, 528 Zhangheng Road, Shanghai 201203, China

## Abstract

This study aims at introducing a method for individual agreement evaluation to identify the discordant raters from the experts' group. We exclude those experts and decide the best experts selection method, so as to improve the reliability of the constructed tongue image database based on experts' opinions. Fifty experienced experts from the TCM diagnostic field all over China were invited to give ratings for 300 randomly selected tongue images. Gwet's AC_1_ (first-order agreement coefficient) was used to calculate the interrater and intrarater agreement. The optimization of the interrater agreement and the disagreement score were put forward to evaluate the external consistency for individual expert. The proposed method could successfully optimize the interrater agreement. By comparing three experts' selection methods, the interrater agreement was, respectively, increased from 0.53 [0.32-0.75] for original one to 0.64 [0.39-0.80] using method A (inclusion of experts whose intrarater agreement>0.6), 0.69 [0.63-0.81] using method B (inclusion of experts whose disagreement score=“0”), and 0.76 [0.67-0.83] using method C (inclusion of experts whose intrarater agreement>0.6& disagreement score=“0”). In this study, we provide an estimate of external consistency for individual expert, and the comprehensive consideration of both the internal consistency and the external consistency for each expert would be superior to either one in the tongue image construction based on expert opinions.

## 1. Introduction

Recently, traditional Chinese medicine (TCM), as a kind of complementary and alternative medicine with a history of five thousand years, has been gradually accepted and embraced by the western medicine system, while, in traditional Chinese medicine, tongue diagnosis plays an important role in the clinical syndrome differentiation and therapeutic evaluation. However, the observation diagnosis of varied tongue features is often biased by the variation of the observers' subjective experience, the uncertainty of the classification standard, and the external lighting environment, so it has become the bottleneck of TCM objectification and internationalization.

Nevertheless, with the spring-up of computer image processing techniques for the past 30 years, tongue image diagnoses have gradually been objectified and quantified, which have greatly promoted the development of TCM diagnosis technology. The digital tongue image database construction based on experts' opinions has become an inevitable trend of the objectification of tongue diagnosis. While the high-quality labeled data is the foundation of the database, in order to obtain the high-quality labeled data to construct a more reliable database based on clinical decision-support from experts, a reliability and agreement evaluation of the obtained data is essential. As for the methods of reliability and agreement study, Kappa statistics, firstly proposed by Cohen in 1960, is used as a scientific indicator of the degree of agreement. Since then, Kappa statistics has been widely used in the clinical agreement and reliability studies for nominal and ordinal measurement, such as in the neurology [1], pathology [2], epidemiology [3], clinical diagnostics especially for medical images [4, 5], and clinical therapeutic evaluation [6]. In addition, according to the different data types and the number of raters, corresponding methods of agreement and reliability assessment are recommended. For example, Cohen's Kappa can be used in nominal data with two raters. Fleiss' Kappa is fit for nominal data for more than two raters. Weighted Kappa can be applied in ordinal data and intraclass correlation coefficients (ICC) are suitable for continuous data [7]. Moreover, the benchmarks for the range of varied agreement coefficients' values are provided by Landis and Koch with 0–0.20 as poor, 0.21–0.40 as fair, 0.41–0.60 as moderate, 0.61–0.80 as substantial, and 0.81–1.0 as almost perfect [8].

Therefore, studies have been carried out to assess the interrater and intrarater agreement for tongue diagnosis among those TCM practitioners [9–12], from which widespread inconsistencies are commonly observed. Besides, there remain some problems in previous studies that need to be discussed. First, these discrepancies among raters have indicated that large-scale agreement studies where many raters contribute ratings should be conducted. Second, the statistical methods used in those studies to assess the agreement and reliability are limited to Kappa coefficient. However, combined with our preliminary study, we found low Kappa values for certain items despite the high percentage of agreement, which has been defined as the “Kappa paradox” by Feinstein and Cicchetti [13], so that the straightforward interpretation of the magnitude for Kappa value may not reflect the true condition. Third, the traditional coefficients to evaluate the interrater agreement are actually total agreement for an experts' group. For expert individual, only intrarater agreement could be obtained to reflect the internal consistency. How to evaluate the external consistency for each expert remains unreported.

To the best of our knowledge, no researches have provided an approach which is appropriate for the construction of a reliable tongue image database based on expert opinions. Thus, through the large-scale agreement study of expert opinions, our study focuses on identifying the discordant raters from the experts' group and if there exist related method to assess the external consistency of individual expert and, in addition, what the best method for experts selection would be in order to construct a more reliable database.

## 2. Methods

### 2.1. Subjects

A total of 300 randomly selected tongue images were collected by TDA-1 hand-held tongue image acquisition device [14] under the same standardized tongue image acquisition process. They were all obtained from the patients from the physical examination center or the outpatient department in Shuguang hospital affiliated to Shanghai University of traditional Chinese medicine during the year 2015. Among them, 35 randomly chosen tongue images were repeated twice to assess the intrarater agreement, because at least this sample size can give a stable evaluation of the intrarater agreement for each rater according to our preliminary research with 10 raters for 60 tongue images.

### 2.2. Ethics Statement

IRB of Shuguang Hospital affiliated with Shanghai University of TCM approved the study (no. 2015-388-16-01), and written informed consent was obtained from all included subjects according to the Declaration of Helsinki.

### 2.3. Instruments

**Figure 1:**
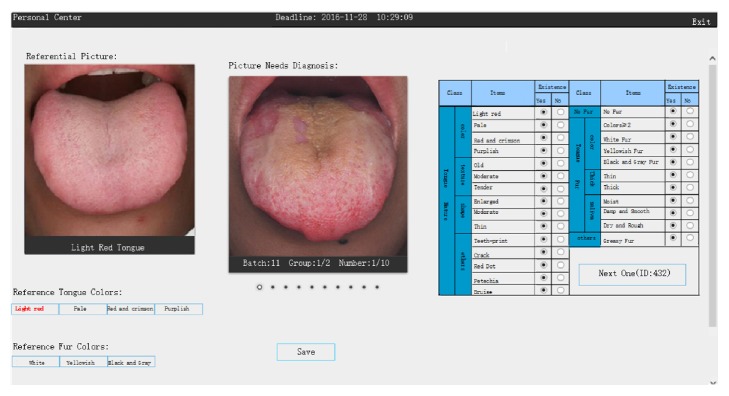
Web interface of tongue image diagnosis for experts.

The ratings for tongue image diagnosis were conducted on a web interface (Figure 1); we created it in order to allow a remote collection of tongue image ratings by the experts from the TCM diagnostic field all over China. All tongue images were converted into 300*∗*300 pixel images for display. The features of those tongue images which needed to be classified by the experts consist of tongue color (pale tongue, light red tongue, red and crimson tongue, and purplish tongue), tongue texture (old, moderate, and tender), tongue shape (enlarged, moderate, and thin), other morphological tongue features (teeth-print, red dot, crack, petechia, and bruise), fur existence (no, yes), fur color (white fur, yellowish fur, black, and gray fur), fur thickness (thin fur, thick fur), fur saliva (moist fur, dry and rough fur, and damp and smooth fur), and other fur features (greasy fur). All the above features were classified into “existed” or “not existed.” In addition, in order to minimize the color bias caused by the different monitors, the referential images for the standardized tongue colors (pale tongue, light red tongue, red and crimson tongue, and purplish tongue) and standard fur colors (white fur, yellowish fur, and the black and gray fur) were provided on the web next to the images which needed to be diagnosed.

### 2.4. Raters

A total of 50 experts who come from the TCM diagnostic field all over China are recruited in this study. They are all registered doctors of TCM who were tested on their ability to identify different tongue features as part of their certification with at least a professional level certificated as “secondary senior.” Experts would receive a small remuneration for their work. Each expert would be assigned an account by administer who was blinded to their rating results, and then experts would enter the tongue image diagnosis system to give ratings for those tongue images features independently.

### 2.5. The Proposed Method

**Figure 2:**
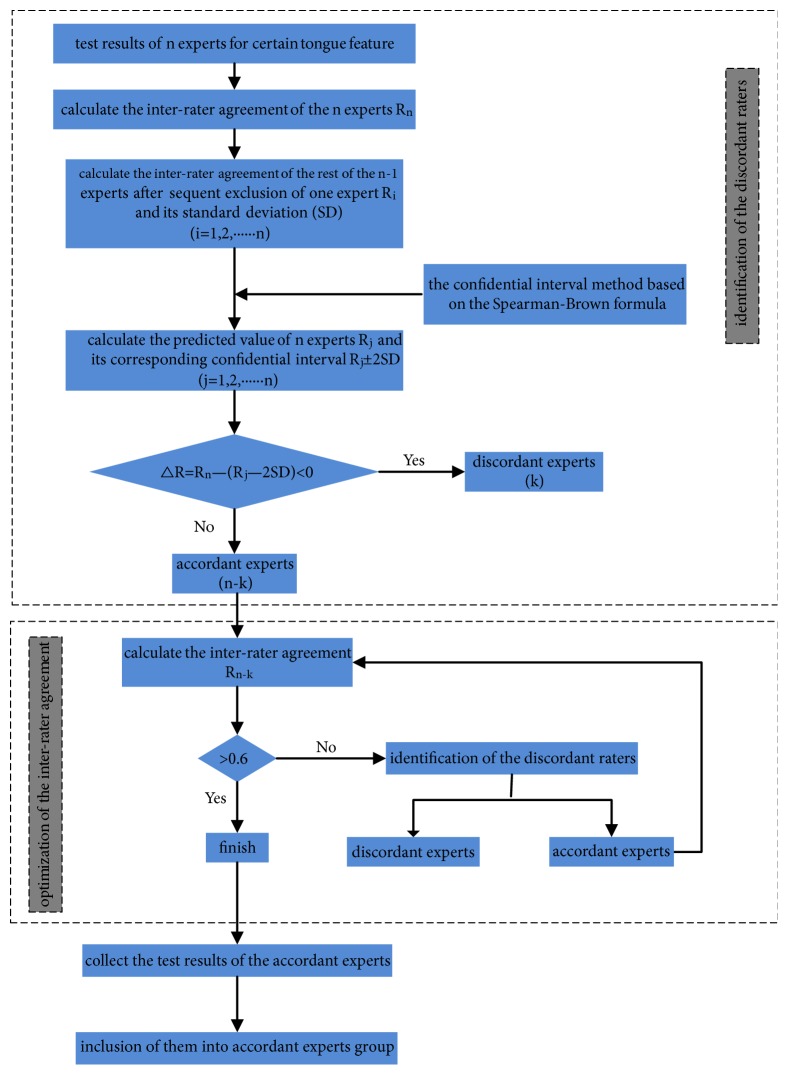
The whole process of the proposed method.

In order to assess the individual agreement for each expert, two procedures were included. One is the identification of the discordant experts; the other is the continued optimization of the interrater agreement. For the former one, the confidential interval method based on the Spearman–Brown formula was used to analyze whether the information provided by one expert was consistent with the whole experts group. This method was brought up by van Ast JF [15] in the reliability study of epileptic seizures diagnosed by neurologists/epileptologists based on intraclass coefficients (ICC). The process to detect the experts with deviating opinions has the following steps: first, use all of the n experts' results to calculate their reliability Rn by Gwet's AC_1_, and then calculate the reliability of the rest of the n-1 experts' results R_i_ (i=1, 2, 3……n) after exclusion of one expert's results in sequence and its corresponding standard deviation (SD). Next, by the application of Spearman–Brown formula and R_i_, the predicted reliability of the n experts R_j_ (j=1, 2, 3……n) and its confidential interval (R_j_±2SD) are calculated. Here, we used R_j_±2SD to calculate the confidential interval, because the detecting process of the experts with deviating opinions from the whole experts' group is similar to the “outliers” identification in the initial group. If the actual reliability for n experts R_n_ falls within the confidential interval of the predicted reliability for n experts R_j_, it suggests that the excluded expert provided consistent opinions, while if R_n_ > R_j_+2SD, it means the excluded expert could improve the reliability of the whole experts group; however if R_n_ < R_j_-2SD, it indicates the excluded expert could decrease the reliability of the whole experts group. In our study, we aim at establishing a more reliable tongue image database; therefore, we only need to find those experts whose results would decrease the whole reliability for all the experts. In other words, suppose △R=R_n_-(R_j_-2SD), for each exclusion of one expert, the corresponding △R would be calculated, and △R<0 is an indication that the excluded expert was inconsistent with the rest of the experts. For the latter procedure, it was conducted at the premise of the former one, and the cut-point for this process was set to 0.6 which suggests that the reliability arrives at a “substantial” level (agreement coefficient > 0.6). We assume that k experts were detected as discordant raters in the procedure of the identification of the inconsistent experts, and then the reliability of the rest of the n-k experts R_n-k_ could be calculated. If R_n-k_ ≤ 0.6, it suggests that the reliability is below the “substantial” level; at this moment, the circular application of procure one could be conducted until the reliability reaches the “substantial” level (R_n-k_ > 0.6) after exclusion of those inconsistent experts or could not be optimized anymore, and this procure could be called the optimization of the interrater agreement. The whole process of this proposed method is shown on Figure 2.

In the process mentioned above, we assume that the recognition of discordant raters was performed m times and k experts were identified and excluded until the reliability of the rest of the n-k experts R_n-k_ > 0.6 (or could not be optimized anymore). Then the individual agreement for the discordant raters who were identified at the first time could be scored “m”; the discordant raters who were identified at the second time could be scored “m-1”… and so on, until the discordant raters who were identified at the m time could be scored “1”; the rest of the n-k experts could be scored “0”; the detailed scoring method is shown in Table 1. According to this scoring method, we can obtain the scores for all the participated experts. Because the score reflects the degree to which one expert disagrees with the whole experts' group, compared with the intrarater agreement to assess the internal consistency of one expert, and this scoring method manifests the external consistency of one expert. The higher the score is, the more the experts disagree with the remaining experts and vice versa. So we call it disagreement score.

Under the above condition, along with the corresponding intrarater agreement, we compared the three experts' selection methods for the standard tongue image database construction, respectively, method A (inclusion of experts who had at least a “substantial” internal consistency with their intrarater agreement>0.6), method B (inclusion of experts who had at least a “substantial” external consistency with their disagreement score=“0”), and method C (inclusion of experts who had at least a “substantial” internal consistency and a “substantial” external consistency with their intrarater agreement>0.6 and disagreement score=“0”).

### 2.6. Data Analysis

According to the literature, Gwet's AC_1_ was used to evaluate the reliability for the experts' ratings, because this method could overcome the “two paradoxes” caused by Kappa statistics and be in-line with the percentage level of agreement [16]. Dunnett's T3 multiple comparison test in ANOVA was used for the pair-wise comparison of the three different expert selection methods for normally distributed data, while Kruskal-Wallis ANOVA was for nonnormally distributed data and a Dunn-Bonferroni test for post hoc pairwise comparisons. For continuous variables, all data were presented as median and interquartile range, and p value less than 0.05 is considered statistically significant. All data were statistically analyzed through the IBM SPSS21.0 (IBM Corporation, Armonk, NY, USA), all related agreement analyses were performed under the AgreeStat 2015 (Advanced analytics, LLC, Gaithersburg, MD, USA), and the optimization process of the interrater agreement was written in Python3.5.

## 3. Results

### 3.1. The Results for the First Identification of Discordant Experts

In the 50 experts' rating results from the 230 nontesting tongue images, the discordant experts for all the 25 tongue features were recognized, and the interrater agreements before and after the removal of those discordant experts are manifested in Table 2, from which we can see that the interrater agreement for all the 25 tongue features were increased after the first exclusion of those identified experts.

### 3.2. The Optimization Results of the Interrater Agreement

After the first recognition and removal of discordant experts from the 50 experts, the reliability for some tongue features was still below the “substantial” level (AC_1_ ≤ 0.6), which included the tongue body features (light red tongue, moderate texture, tender tongue, enlarged tongue, moderate tongue shape, teeth-print, and crack) and tongue fur features (white fur, thin fur, thick fur, moist fur, and greasy fur). Among the above tongue features, the interrater agreement of the experts' classification for “moderate tongue texture” was the lowest (AC_1_ value=0.0948). We took it as an example, and the results for the optimization process of the interrater agreement are manifested in Table 3. From this table, 8 recognition times were conducted to identify 34 discordant experts, which improved the interrater agreement from 0.0948 to 0.6333. Similarly, the results for optimization of the interrater agreement for all the above tongue features are shown in Table 4, from which we could see interrater agreement for all tongue features could be successfully optimized to the “substantial” level (AC_1_ value>0.6) except for “thin fur” whose largest optimized value was 0.4601.

### 3.3. The Individual Agreement for 50 Experts

**Figure 3:**
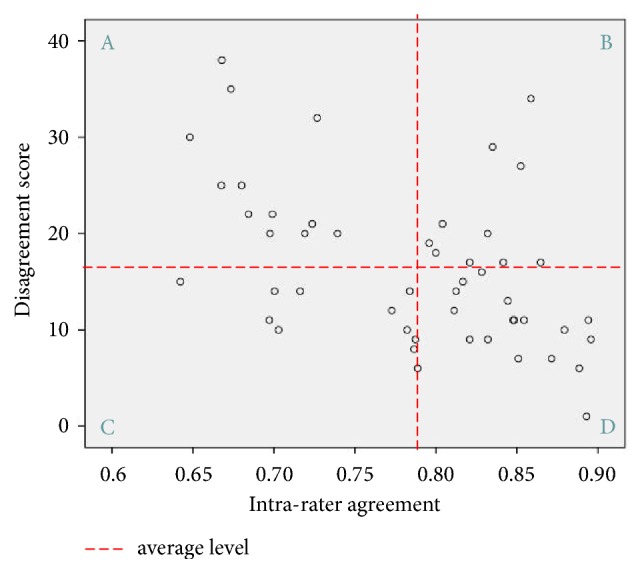
Distribution of interrater agreement and disagreement scores for 50 experts.* Notes*. Section A= experts with lower internal consistency and more discordant test results; Section B= experts with higher internal consistency but more discordant test results; Section C= experts with lower internal consistency but less discordant test results; Section D= experts with higher internal consistency and less discordant test results.

Taking “the moderate texture” as an example, 8 recognition times with 34 discordant experts were progressed, the disagreement scores for those discordant experts are shown in Table 5, and the disagreement scores for the rest of the experts apart from the deviating ones were scored “0.” In this way, the external consistency of each expert for all the 25 tongue features could be indicated by the disagreement scores. Eventually the disagreement scores and intrarater agreement for 50 experts are shown in Figure 3, in which the red dashed line represented the average level for all the 50 experts, and they divided the scatter diagram into 4 sections (A, B, C, and D), most experts were distributed in sections A and D, which suggests that the experts with a higher internal consistency usually tend to have more consistent test results with the remaining ones, while there are still certain experts whose test results may disagree (agree) with the rest of the ones even though they have a relatively higher (lower) internal consistency.

### 3.4. The Interrater Agreement Results of Different Expert Selection Methods

**Figure 4:**
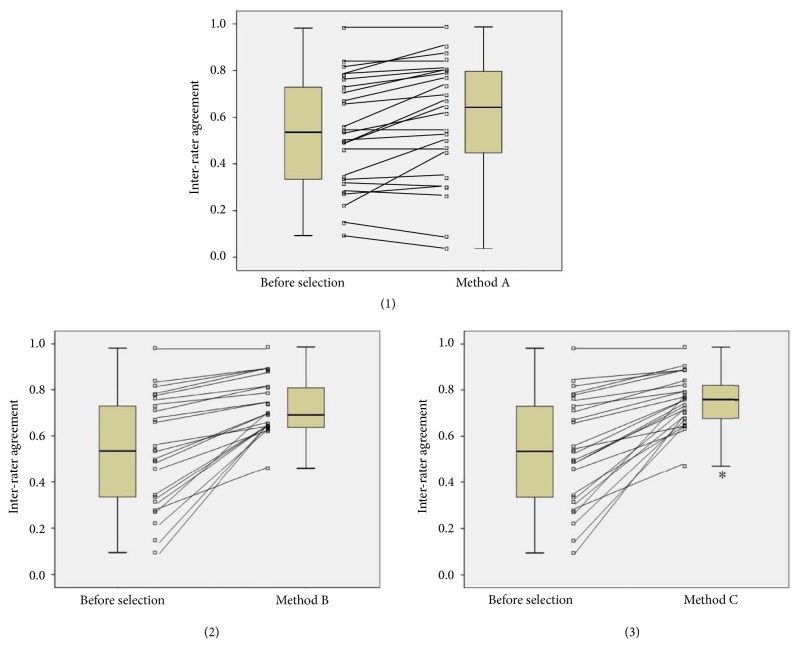
Interrater agreement of 25 tongue features after three expert selection methods.* Notes*. *∗* compared with interrater agreement before selection, P<0.05.

According to the above individual agreement results of the 50 experts for the 25 tongue features, we further compared the interrater agreement results of three different expert selection methods for the 230 nontesting tongue images. The effects of these three different expert selection methods on the interrater agreement are shown in Figure 4, from which we could see that, through the application of these three expert selection methods, the interrater agreements were all increased compared to the original one before selection (0.53 [0.32-0.75]). Among them, method C had the best performance (0.76 [0.67-0.83]), method B took second place (0.69 [0.63-0.81]), and method A was the worst (0.64 [0.39-0.80]). However, statistical significance was only found in the pairwise comparison between method C and the original one before selection (P<0.05). Based on the above results, it indicates that, in the three selection methods for the expert, the comprehensive consideration of both the internal consistency and the external consistency for each expert would be superior to either one.

## 4. Discussion

In the progress of the tongue image database construction based on expert opinions, the main problem is how to assess the agreement or reliability for each expert, so that we could select the appropriate ones to establish a more reliable database based on experts' clinical decision-support. However, for individual rater, only intrarater agreement could be evaluated, and the traditional agreement coefficients for interrater agreement such as intraclass correlation (ICC), Kappa coefficient, and AC_1_ value are actually measurements of total agreement that reflect the whole reliability condition for a group of raters. Some researchers made some attempts on the above issues; however they were limited to certain data type (continuous, ordinal, and nominal). For example, Barnhart put up coefficient of individual agreement (CIA) to assess individual agreement for the continuous data [17, 18]. Nelson raised an approach based upon the class of generalized linear mixed models to assess the influence of rater and subject characteristics on measures of agreement for ordinal ratings [19]. Ruddat proposed a Kappa-based method to improve assessing agreement between several observers [20]. Different from the above studies, we proposed the optimization method of interrater agreement on the basis of the available confidential interval method based on the Spearman–Brown formula and further introduced the disagreement scores to evaluate the degree to which the experts were inconsistent with the rest of the experts. Besides, the method provided in our study is not bound by the data type limit because it is based on value of certain agreement coefficient itself, so it could be widely applied to varied agreement coefficients for different data type (ordinal, nominal, or continuous) in other standard knowledgebase construction based on clinical decision-support from experts.

In this study, we first verified the effectiveness of the available confidential interval method based on the Spearman–Brown formula for the identification of discordant experts (Table 2). Besides, the optimization method of interrater agreement was conducted for tongue features whose interrater agreements were still less than or equal to 0.6 after the first identification of the discordant experts. And the optimizing results of interrater agreement for these tongue features are manifested in Table 4, which indicates that the interrater agreement for all tongue features could be obviously optimized by circular identification process of the inconsistent experts.

Furthermore, along with the intrarater agreement for the internal consistency for individual expert, the disagreement score was introduced to assess the external consistency of each expert. Then, we studied the relationship between the internal consistency indicated by intrarater agreement and the external consistency suggested by disagreement scores for those participant 50 experts (Figure 3).The results illustrate that the experts with a higher internal consistency usually tend to have more external consistency. However, there are still some experts whose internal consistency was not corresponding to their external consistency.

Therefore, we further compared different expert selection methods for 25 tongue features (Figure 4).The results indicate that, in the standard tongue image database construction based on the expert opinions, both the experts' internal consistency (indicated by intrarater agreement) and their external consistency (indicated by disagreement scores) should be considered to get a more reliable database based on clinical decision-support rather than either one.

Last but not the least, this research was conducted on a web interface which could allow a remote collection of tongue image ratings by involving as many raters as possible to participate in this research simultaneously and independently. In spite of the above merits, a different result might be obtained if the ratings were not conducted on a web interface, and color difference is the key problem which might lead to it. However, in this research, we designed the following contents to avoid this problem as much as possible: Firstly, all the tongue images were acquired by TDA-1 hand-held tongue image acquisition device, which we mentioned before in our other paper [14]. By imitating a stable illumination environment which is closest to the natural light in the traditional visual observation, the standardized tongue image acquisition process can guarantee a more realistic color feature for the tongue image. Secondly, in order to minimize the color bias caused by the different monitors that experts used, the referential images for the standardized tongue colors (pale, light red, red and crimson, and purplish) and standard fur colors (white, yellowish, the black, and gray) were provided on the web next to the images which needed to be diagnosed.

## 5. Conclusion

In this study, we successfully optimize the interrater agreement and provide an estimate of external consistency for individual expert. Besides, we find that the comprehensive consideration of both the internal consistency and the external consistency for each expert would be superior to either one in the tongue image construction based on expert opinions.

## Figures and Tables

**Table 1:** Disagreement scoring method for each expert.

experts	recognition times	disagreement scores
discordant experts (K)	1	m
	2	m-1
	3	m-2
	⋮	⋮
	⋮	⋮
	m	1

rest of the experts (n-k)	none	0

**Table 2:** The inter-rater agreement changes after the first identification of discordant experts.

25 features		inter-rater agreement	
		before first identification	after first identification
tongue body	pale	0.7622	0.8125
	light red	0.3431	0.4357
	red and crimson	0.5397	0.6254
	purplish	0.7292	0.7845
	old	0.5566	0.6420
	moderate texture	0.0948	0.1674
	tender	0.5349	0.5842
	enlarged	0.3345	0.4411
	moderate shape	0.1477	0.2212
	thin	0.8389	0.8864
	teeth-print	0.4580	0.5349
	red dot	0.4911	0.6269
	crack	0.4972	0.5686
	bruise	0.7780	0.8787
	petechia	0.7803	0.8897

tongue fur	white fur	0.4902	0.5941
	yellowish fur	0.6699	0.7398
	black and gray fur	0.9818	0.9869
	white and yellowish fur	0.8173	0.8826
	thin fur	0.2769	0.3507
	thick fur	0.3147	0.3816
	moist fur	0.2706	0.3625
	damp and smooth fur	0.7115	0.8084
	dry and rough fur	0.6600	0.7357
	greasy fur	0.2213	0.3167

**Table 3:** Improving process of the interrater agreement for “the moderate tongue texture.”

Recognition times	Identified discordant experts	Rest experts	Inter-rater agreement for the rest of the experts
1	rater 45, rater 49, rater 32, rater 42, rater 34, rater 40	44	0.1674
2	rater 20, rater 16, rater 13, rater 3	40	0.2220
3	rater 5, rater 43	38	0.2470
4	rater 15, rater 25	36	0.2707
5	rater 29, rater 39, rater 33, rater 47	32	0.3247
6	rater 31, rater 35, rater 50, rater 26, rater 38	27	0.4098
7	rater 41, rater 17, rater 1, rater 10	23	0.4890
8	rater 46, rater 19, rater 44, rater 37, rater 30, rater 18, rater 11	16	0.6333

**Table 4:** Optimizing results of interrater agreement for all the tongue features which needed optimization.

Optimized tongue features		recognition times	rest of the experts	Inter-rater agreement	
				Before optimization	After optimization
Tongue body	Light red	3	19	0.3431	0.6450
	moderate texture	8	16	0.0948	0.6333
	tender	2	41	0.5349	0.6535
	Enlarged	3	26	0.3345	0.6404
	Moderate shape	5	10	0.1477	0.6310
	Teeth-print	2	16	0.4580	0.6363
	crack	2	21	0.4972	0.6960

Tongue fur	White fur	2	35	0.4902	0.6907
	Thin fur	3	24	0.2769	0.4601
	Thick fur	3	4	0.3147	0.6406
	Moist fur	5	22	0.2706	0.6989
	Greasy fur	4	24	0.2213	0.6200

**Table 5:** Disagreement scores of discordant experts for “the moderate tongue texture”.

Recognition times	Identified discordant experts	The disagreement scores
1	rater 45, rater 49, rater 32, rater 42, rater 34, rater 40	8
2	rater 20, rater 16, rater 13, rater 3	7
3	rater 5, rater 43	6
4	rater 15, rater 25	5
5	rater 29, rater 39, rater 33, rater 47	4
6	rater 31, rater 35, rater 50, rater 26, rater 38	3
7	rater 41, rater 17, rater 1, rater 10	2
8	rater 46, rater 19, rater 44, rater 37, rater 30, rater 18, rater 11	1

## Data Availability

The datasets generated and analyzed during the current study are not publicly available due to the confidentiality of the data, which is an important component of the National Key Technology R&D Program of the 13th Five-Year Plan (no. 2017YFC1703301) in China, but are available from the corresponding author on reasonable request.
